# Deletion of a polyketide synthase *phoE* induces monoterpenes production in ascidian-derived fungus *Diaporthe* sp. SYSU-MS4722

**DOI:** 10.3389/fmicb.2025.1593027

**Published:** 2025-07-03

**Authors:** Siwen Yuan, Shitao Zhuang, Yanhong Han, Yuhang Wen, Senhua Chen, Lan Liu, Qifeng Lin, Yan Yan, Zhizeng Gao, Qilin Wu

**Affiliations:** ^1^School of Bioengineering, Zunyi Medical University, Zhuhai, China; ^2^Guangdong Provincial Engineering Research Center, The Fifth Affiliated Hospital, Sun Yat-sen University, Zhuhai, Guangdong, China; ^3^School of Marine Sciences, Sun Yat-sen University, Zhuhai, China; ^4^Shenzhen Lions King Hi-Tech Co., Ltd., Shenzhen, China

**Keywords:** genome mining, monoterpenes, polypropionate derivatives, phomoxanthone A, antiinflammatory activity

## Abstract

Marine-derived fungi represent an underexplored source of monoterpenes, with only sporadic reports over the past two decades, making their discovery particularly challenging. Whole-genome sequencing of *Diaporthe* sp. SYSU-MS4722, an ascidian-derived fungus isolated from *Styela plicata*, identified 130 biosynthetic gene clusters (BGCs), underscoring its vast biosynthetic potential. However, previous studies demonstrated that *Diaporthe* sp. SYSU-MS4722 predominantly produces xanthone phomoxanthone A under standard laboratory conditions, suggesting that many BGCs remain cryptic. Notably, 16 of these BGCs encode terpene synthase genes, indicating the potential for monoterpene biosynthesis. To activate these silent BGCs and discover monoterpenes, we deleted the polyketide synthase (PKS) gene *phoE*, responsible for phomoxanthone A biosynthesis, generating the mutant strain *Diaporthe* sp. SYSU-MS4722Δ*phoE*, from which three new monoterpenes, diaporterpenes D-F (*1-3*), three known monoterpenes (**4-6**), and two new polypropionate derivatives, diaporpolypropionate A (**7**) and diaporpolypropionate B (**8**), were isolated. Compounds **1**, **4**, **7**, and **8** were evaluated for their cytotoxicity and anti-inflammatory effects in human non-small cell lung cancer A549 cells. Compound **1** exhibited cytotoxic activity with an IC_50_ value of 89.33 μM. Compounds 4 and 7 demonstrated anti-inflammatory activity, as measured by an ELISA assay assessing the inhibition of IL-6 secretion, with EC_50_ values of 41.85 μM and 70.80 μM, respectively. These results underscore genome mining as a powerful tool in natural product discovery and the exploration of novel chemical space from marine fungal resources.

## 1 Introduction

Fungal-derived natural products have played a crucial role in the discovery and development of a diverse array of bioactive compounds (Berdy, 2012; Keller, 2015). However, isolating many of these natural products remains challenging, as numerous biosynthetic gene clusters (BGCs) remain cryptic under typical laboratory conditions. Therefore, activating these silent BGCs to enable the production of diverse secondary metabolites has become a critical challenge in natural product research (Brakhage, 2013). Studies have shown that one-strain-many-compounds (OSMAC) (Scherlach and Hertweck, 2006; Scherlach et al., 2010), co-culturing with different microorganisms (Brakhage, 2013; Schroeckh et al., 2009), epigenetic approaches (Zheng et al., 2017; Fan et al., 2017), heterologous expression (Biggins et al., 2011; Yuan et al., 2022a), and metabolic shunting activation can effectively activate silent BGCs (Wei et al., 2021; Ning et al., 2024), leading to the production of diverse new compounds.

In marine fungal natural products, monoterpenes are particularly rare but represent an important class of natural products. To date, total 15 new monoterpenes have been isolated from marine-derived fungi, including penicipenes A and B (Cheng et al., 2019), nectriapyrones C and D (Gong et al., 2015), 2-(2-hydroxy-4-methylcyclohex-3-enyl) propanoic acid (Liu et al., 2017), eutypellol B (Liu et al., 2017), pestalotiolactones C and D (Li et al., 2019), 1-O-(α-D-mannopyranosyl) geraniol (Yun et al., 2015), (1*S*,2*S*,3*S*,4*R*)-3-chloro-4-(2-hydroxypropan-2-yl)-1-methylcyclohexane-1,2-diol (Huang et al., 2006), (7*S*) and(7*R*)-1hydroxy-3-*p*-menthen-9-oic acids (Song et al., 2018), and diaporterpenes A-C (Zhai et al., 2022). Discovering new monoterpenes from marine-derived fungi remains a challenging task.

**FIGURE 1:**
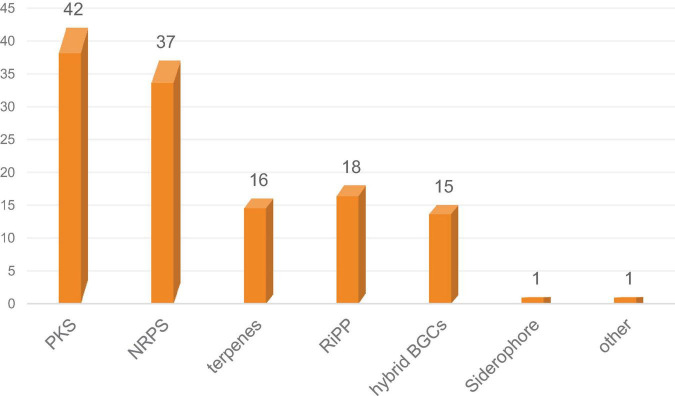
Distribution and classification of BGCs predicted in *Diaporthe* sp. SYSU-MS4722.

**FIGURE 2:**
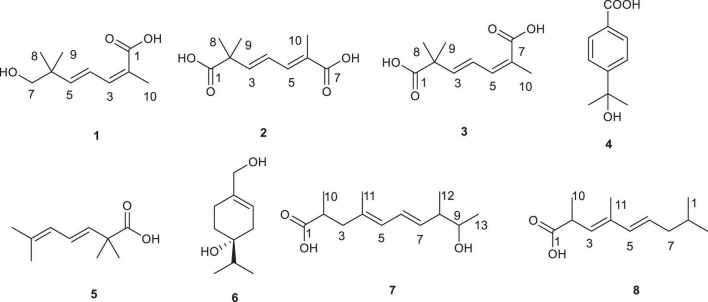
Chemical structures of compounds **1**–**8**.

*Diaporthe* sp. SYSU-MS4722, an ascidian-derived fungus isolated from *Styela plicata*, produces bioactive natural products, including polyketides and monoterpenes. Notably, it primarily biosynthesizes substantial quantities of phomoxanthone A (Supplementary Figure S1). In contrast, the production of monoterpenes, diaporterpenes A–C, is remarkably low under typical laboratory conditions (Supplementary Figure S2) (Zhai et al., 2022; Chen et al., 2022). Whole-genome sequencing and BGCs analysis identified 130 BGCs, highlighting its extensive biosynthetic potential. Among these, 16 BGCs encode terpene synthase genes, suggesting the capability for monoterpene biosynthesis (Figure 1). To further explore monoterpene production in this strain, we generated the mutant strain *Diaporthe* sp. SYSU-MS4722*△phoE* by deleting the PKS gene *phoE*, which is essential for phomoxanthone A biosynthesis. As a result, the mutant strain lost the ability to produce phomoxanthone A, thereby redirecting metabolic flux toward alternative biosynthetic pathways. Cultivation of this mutant in rice medium enabled comprehensive chemical profiling, leading to the discovery of three new monoterpenes, diaporterpenes D-F (**1**–**3**), three known monoterpenes (**4**–**6**), and two new polypropionate derivatives, diaporpolypropionate A (**7**) and diaporpolypropionate B (**8**) (Figure 2). Herein, we report the details of the isolation, structural elucidation, and bioactivity of compounds **1**-**8**.

## 2 Materials and methods

### 2.1 General materials

Chemicals were purchased from Sangon Biotech Co., Ltd., Thermo Fisher Scientific, Sigma-Aldrich, or J&K Scientific Ltd., unless otherwise stated. Column chromatography (CC) was performed using silica gel (200-300 mesh, Qingdao Marine Chemical Factory, Qingdao, China). Optical rotations were recorded on an MCP-200 polarimeter (Anton Paar, Graz, Austria). Infrared (IR) spectra were recorded on a Fourier-transform infrared spectrometer coupled with an EQUINOX 55 infrared microscope (Bruker, Fällanden, Switzerland). UV spectra were obtained using a Waters 2998 photodiode array detector (Waters, Boston, United States). High-resolution electrospray ionization mass spectrometry (HRESIMS) data were acquired on an LTQ-Orbitrap LC-MS spectrometer (Thermo Fisher Scientific, MA, United States). NMR spectra were recorded on a Bruker Avance 400 MHz spectrometer (Bruker, Switzerland) using tetramethylsilane (TMS) as the internal standard. Semi-preparative and analytical HPLC were performed on a Waters 1525 system equipped with a Waters 2998 photodiode array detector (Waters, Boston, United States), using a Welch Ultimate^®^ XB-C18 column (10 mm × 250 mm, 5 μm, Welch Materials, Inc., Shanghai, China) and a COSMOSIL 5C18-AR-II column (4.6 mm × 250 mm, 5 μm, Nacalai Tesque, Inc., Kyoto, Japan), respectively.

### 2.2 Strains and media

The strain *Diaporthe* sp. SYSU-MS4722 was isolated from the ascidian *Styela plicata* collected from the Bay of Da’ao, Shenzhen City, Guangdong Province, China, in April 2016. Fungal isolation followed a standard protocol (Raja et al., 2017), and fungal identification was confirmed by DNA amplification and sequencing of the ITS region. The sequence has been deposited in GenBank (accession no. OK623372), and BLAST analysis showed it to be 100% identical to *Diaporthe* sp. NFIF-2-6 (MW202988.1). Detailed information about the fungus was previously described (Yuan et al., 2022b,Zhai et al., 2022; Chen et al., 2022).

The mutant strain *Diaporthe* sp. SYSU-MS4722*△phoE*, in which the *phoE* gene encoding phomoxanthone A PKS has been deleted, was successfully generated in our previous study (Supplementary Figure S3) (Yuan et al., 2022b). The mutant strain and wild-type strain *Diaporthe* sp. SYSU-MS4722 were first grown in 200 mL of PDB medium at 30°C for 3 days to establish seed cultures, which were then transferred to rice medium containing 50 g of rice and 3% sea salt.

### 2.3 Genome sequencing and annotation

Genomic DNA of *Diaporthe* sp. SYSU-MS4722 was sequenced on the Illumina HiSeq™ platform with 2 × 150 bp paired-end reads. Raw reads were quality-filtered using Trimmomatic and evaluated by FastQC. High-quality reads were assembled *de novo* using SPAdes, followed by gap filling (GapFiller) and sequence correction (PrInSeS-G). Gene prediction was performed using Prokka, and functional annotation was conducted *via* BLAST+ against NR, SwissProt, TrEMBL, PFAM, and KEGG databases (Koren et al., 2017; Bankevich et al., 2012; Boetzer and Pirovano, 2012). BGCs were predicted using antiSMASH versions 7.0 and 8.0 (Blin et al., 2025; Blin et al., 2023).

### 2.4 Extraction and isolation

The mutant strain *Diaporthe* sp. SYSU-MS4722*△phoE* was fermented in rice medium for 40 days. The mycelium was then sonicated in methanol and the extract was concentrated under reduced pressure at 45°C, yielding a total extract of 120 g. After dissolving the total extract in 100 mL of H_2_O, it was extracted three times with an equal volume of EtOAc, and the solvent was removed under reduced pressure to give the EtOAc extract (50 g), which was then subjected to silica gel column chromatography with ethyl acetate/petroleum ether mixtures at 20, 40, 60, and 100% EtOAc. The 40% EtOAc fraction was further separated by reverse-phase silica gel chromatography with a gradient of 40, 60, 80, and 90% MeOH/H_2_O, yielding fractions Fr.1-Fr.4. Fr.1 was purified by reverse-phase HPLC using 20% MeCN/H_2_O and then further separated on a Sephadex LH-20 column (CH_2_Cl_2_/MeOH 1:1), followed by HPLC with 25% MeCN/H_2_O to isolate **1** (3.6 mg) and a pair of chiral isomers. This chiral mixture was separated using a chiral column (Ultimate Amy-SR, MeCN/H_2_O = 15/85, flow rate = 3 mL/min), yielding **2** (2.0 mg) and **3** (1.6 mg).

Fr.2 was first separated on a Sephadex LH-20 column (CH_2_Cl_2_/MeOH 1:1) and then subjected to reverse-phase HPLC with 30% MeCN/H_2_O to yield Fr.2.1 and Fr.2.2. Fr.2.1 was further purified using reverse-phase HPLC with 30% MeCN/H_2_O, yielding **7** (2.8 mg) and **8** (2.6 mg). Fr.2.2 was separated by silica gel (petroleum ether/EtOAc 3:1) and then purified by reverse-phase HPLC with 35% MeCN/H_2_O, yielding **4** (3.8 mg), **5** (1.8 mg), and **6** (1.5 mg).

Diaporterpenes D (**1**): colorless oil; ^1^H NMR (600 MHz, Methanol-*d*_4_) δ 7.06 (H-4, dd, *J* = 15.6, 11.0 Hz, 1H), 6.38 (H-3, d, *J* = 10.9 Hz, 1H), 5.88 (H-5, d, *J* = 15.6 Hz, 1H), 3.31 (H-7, s, 2H), 1.93 (H-10, s, 3H), 1.03 (H-8, 9, s, 6H).^ 13^C NMR (150 MHz, Methanol-*d*_4_) δ 172.46 (C-1), 148.08 (C-5), 140.35 (C-3), 127.81 (C-2), 126.76 (C-4), 72.09 (C-7), 39.82 (C-6), 24.26 (C-8, 9), 21.11 (C-10). HR-ESIMS *m/z* 207.0989 [M+Na]^+^ (calcd for C_10_H_16_O_3_Na, 207.0992).

Diaporterpenes E (**2**): colorless oil; ^1^H NMR (600 MHz, Methanol-*d*_4_) δ 7.15 (H-5, d, *J* = 11.1 Hz, 1H), 6.44 (H-4, dd, *J* = 15.4, 11.1 Hz, 1H), 6.29 (H-3, d, *J* = 15.4 Hz, 1H), 1.91 (H-10, s, 3H), 1.33 (H-8, 9, s, 6H). ^13^C NMR (150 MHz, Methanol-*d*_4_) δ 181.27 (C-1), 172.94 (C-7), 150.45 (C-3), 140.91 (C-5), 126.81 (C-6), 123.83 (C-4), 46.59 (C-2), 25.76 (C-8, 9), 12.94 (C-10). HR-ESIMS *m/z* 221.0783 [M+Na]^+^ (calcd for C_10_H_14_O_4_Na, 221.0784).

Diaporterpenes F (**3**): colorless oil; ^1^H NMR (600 MHz, Methanol-*d*_4_) δ 7.06 (H-4, dd, *J* = 15.5, 11.1 Hz, 1H), 6.29 (H-5, d, *J* = 11.1 Hz, 1H), 6.03 (H-3, d, *J* = 15.5 Hz, 1H), 1.93 (H-10, s, 3H), 1.30 (H-8, 9, s, 6H). ^13^C NMR (150 MHz, Methanol-*d*_4_) δ 181.12 (C-1), 173.87 (C-7), 144.32 (C-3), 137.56 (C-5), 126.78 (C-4, 6), 45.97 (C-2), 25.74 (C-8, 9), 21.28 (C-10). HR-ESIMS *m/z* 221.0782 [M+Na]^+^ (calcd for C_10_H_14_O_4_Na, 221.0784).

Diaporpolypropionate A (**7**) : colorless oil; ${\left[\alpha \right]}_{\text{D}}^{20}$ + 3.47 (*c* 0.44, MeOH); IR (neat) ν_max_ 3406, 2513 1699, 1456 cm^–1^; ^1^H NMR (600 MHz, Methanol-*d*_4_) δ 6.27 (H-6, dd, *J* = 15.1, 10.7 Hz, 1H), 5.82 (H-5, d, *J* = 10.7 Hz, 1H), 5.51 (H-7, dd, *J* = 15.1, 8.3 Hz, 1H), 3.55 (H-9, p, *J* = 6.5 Hz, 1H), 2.61 (H-2, p, *J* = 6.5, 5.9 Hz, 1H), 2.41 (H-3, dd, *J* = 13.6, 7.3 Hz, 1H), 2.18 (H-8, m, 1H), 2.09 (H-3, dd, *J* = 13.6, 7.5 Hz, 1H), 1.74 (H-11, s, 3H), 1.10 (H-13, H-10, overlap, m, 6H), 1.03 (H-12, dd, *J* = 6.8, 2.5 Hz, 3H). ^13^C NMR (150 MHz, Methanol-*d*_4_) δ 182.35 (C-1), 136.23 (C-7), 134.73 (C-4), 128.26 (C-5), 127.67 (C-6), 72.41 (C-9), 45.94 (C-8), 45.16 (C-3), 39.40 (C-2), 20.78 (C-10 or 13), 17.23 (C-13 or 10), 16.82 (C-12), 16.33 (C-11). HR-ESIMS *m/z* 225.1499 [M-H]^–^ (calcd for C_13_H_21_O_3_, 225.1496).

Diaporpolypropionate B (**8**) : colorless oil; ${\left[\alpha \right]}_{\text{D}}^{20}$ + 1.86 (*c* 0.17, MeOH); IR (neat) ν_max_ 3385, 2503, 1647 cm^–1^; ^1^H NMR (600 MHz, Methanol-*d*_4_) δ 6.07 (H-5, d, *J* = 15.3 Hz, 1H), 5.63 (H-6, dd, *J* = 15.3, 7.5 Hz, 1H), 5.39 (H-3, d, *J* = 9.3 Hz, 1H), 3.66 (H-9, m, 1H), 3.39 (H-2, m, 1H), 2.28 (H-7, m, 1H), 1.90 (H-7, m, 1H), 1.78 (H-11, s, 3H), 1.51 (H-8, m, 1H), 1.22 (H-10, d, *J* = 8.7 Hz, 3H), 1.13 (H-13, d, *J* = 6.4 Hz, 3H), 0.88 (H-12, d, *J* = 7.0 Hz, 3H). ^13^C NMR (150 MHz, Methanol-*d*_4_) δ 181.29 (C-1), 137.07 (C-5), 135.56 (C-4), 131.37 (C-3), 128.40 (C-6), 71.39 (C-9), 41.62 (C-2, C-8), 37.32 (C-7), 20.32(C-13), 19.05 (C-10), 14.65 (C-12), 12.87 (C-11). HR-ESIMS *m/z* 225.1497 [M-H]^–^ (calcd for C_13_H_21_O_3_, 225.1496).

### 2.5 Cytotoxicity assay

Ham’s F-12K complete medium was purchased from China Procera Life Technology Co., Ltd. Dimethyl sulfoxide (DMSO) and fetal bovine serum (FBS) were acquired from Sigma-Aldrich (St. Louis, MO, United States) and BioChannel (BC-SE-FBS07), respectively. The F-12K basal culture medium (iCell-0007) and Calcein / PI Live/Dead Assay Kit (C2015S) were obtained from iCell and Beyotime.

A549 cells were plated in 96-well plates at a density of 7,500 cells per well and exposed to the test compounds at concentrations ranging from 1.5625 to 400 μM. Cell viability was assessed *via* Calcein/Pl staining, with live cells fluorescing green, and monitored in real time using IncuCyte imaging over 48 h. The number of viable (green-stained) cells was quantified.

### 2.6 Anti-inflammatory assay

Human non-small cell lung cancer A549 cells, sourced from Shanghai SIBCB (iCell) Life Sciences & Technology Co., Ltd., China, were cultured in F12K medium (Gibco), supplemented with 10% fetal bovine serum (FBS), 100 IU/mL penicillin, and 100 μg/mL streptomycin (Gibco). The cultures were maintained under standard conditions of 37°C with 5% CO_2_.

The anti-inflammatory effects of the compounds were evaluated using an ELISA assay. Briefly, serial dilutions of each compound, at concentrations below their respective IC_50_ values (ranging from 80 to 160 μM with 8 serial dilutions), were applied to A549 cells for 1 h, followed by the addition of 1 ng/mL rh IL-1β to stimulate IL-6 secretion. After 48 h of incubation at 37°C with 5% CO_2_, supernatants were collected, centrifuged, and IL-6 levels were measured. For the ELISA assay, 50 μL of capture antibody (diluted 1:250 in coating buffer) was added to a 96-well plate and incubated overnight at 4°C. The following day, the plate was washed three times with wash buffer and blocked with 300 μL of blocking buffer per well, incubating for 1 h at room temperature. After washing, standards and samples were added and incubated for 2 h. Detection antibody and HRP-conjugated secondary antibody (both diluted 1:250) were then applied and incubated for 1 h in the dark. Color development was achieved using TMB substrate, and after 10 min, the reaction was stopped with H_2_SO_4_. Absorbance was measured at 450 nm, and the EC_50_ values for each compound were calculated based on the inhibition of IL-6 secretion.

## 3 Results and discussion

### 3.1 Genome and BGC analysis

Illumina sequencing of *Diaporthe* sp. SYSU-MS4722 yielded approximately 12.2 million high-quality paired-end reads. De novo assembly produced 1,220 contigs with a total genome size of 56.97 Mb, an N50 of 120,907 bp, and a GC content of 53.0%. A total of 17,189 protein-coding genes were predicted, along with 112 tRNA and 39 rRNA genes. Functional annotation assigned 94.21% of genes to at least one database, including NR, SwissProt, TrEMBL, PFAM, GO, KEGG, COG, and KOG. Genome mining of *Diaporthe* sp. SYSU-MS4722 using antiSMASH identified 130 putative BGCs, covering diverse classes including PKS, nonribosomal peptide synthetase (NRPS), terpenes, RiPP-like clusters, and hybrid BGCs. Among them, 16 clusters were predicted to be involved in terpene biosynthesis, accounting for approximately 12.3% of the total BGCs. Some terpene clusters displayed homology to known metabolites, including PR-toxin (50%), Culmorin/(+)-Juniperol/15-acetyldeoxynivalenol (100%), and Squalestatin S1 (40%). The remaining terpene clusters showed no close similarity to known references, indicating potential for novel terpenoid biosynthesis.

### 3.2 Fungal fermentation and structure elucidation of new compounds

The mutant strain, *Diaporthe* sp. SYSU-MS4722*△phoE*, was cultured on a solid rice medium at room temperature for 40 days. The metabolites were extracted first with MeOH, followed by continuous extraction with EtOAc to obtain crude metabolites. The crude extract was then subjected to repeated silica gel chromatography and semipreparative HPLC, which led to the isolation of three new monoterpenes, diaporterpenes D-F (**1**–**3**), three known monoterpenes (**4**–**6**), along with two new polypropionate derivatives, diaporpolypropionate A (**7**) and diaporpolypropionate B (**8**).

**FIGURE 3:**
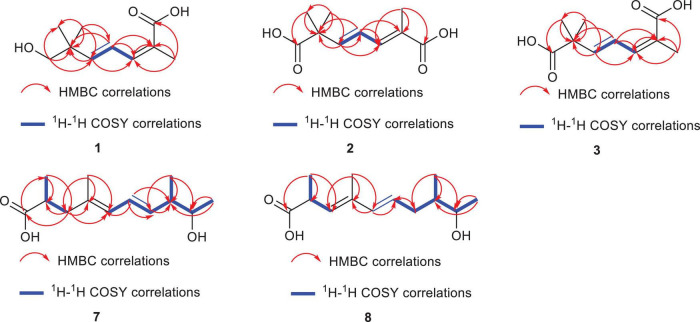
The key HMBC (red arrow) and ^1^H-^1^H correlations of compounds **1–3, 7, 8**.

Diaporterpene D (**1**) is a colorless oil. HR-ESIMS analysis gave an m/z 207.0989 [M+Na]^+^ (calcd for C_10_H_16_O_3_Na, 207.0992), confirming its molecular formula as C_10_H_16_O_3_, with an unsaturation degree of 3. The structure was elucidated based on ^1^H NMR, ^13^C NMR, and HSQC spectra, which revealed the presence of one carbonyl carbon (δ_C_ 172.5), two double bonds [δ_H_ 7.06 (H-4, dd, *J* = 15.6, 11.0 Hz, 1H), δ_C_ 126.8; δ_H_ 6.38 (H-3, d, *J* = 10.9 Hz, 1H), δ_*C*_ 140.4; δ_H_ 5.88 (H-5, d, *J* = 15.6 Hz, 1H), δ_C_ 148.1, δ_*C*_ 127.8], two quaternary carbons (δ_C_ 39.8, 127.8), one methylene group [δ_H_ 3.31 (H-7, s, 2H), δ_C_ 72.1], and three methyl groups [δ_H_ 1.93 (H-10, s, 3H), δ_C_ 21.1; δ_H_ 1.03 (H-8,9, s, 6H), δ_C_ 24.3] (Table 1). Combining the ^1^H-^1^H COSY and ^1^H NMR data indicated the presence of a 1,3-diene fragment in compound **1**. In the HMBC spectrum, H-10 (δ_H_ 1.93) showed correlations with C-1 (δ_C_ 172.5), C-2 (δ_C_ 127.8), and C-3 (δ_C_ 140.4), confirming the connection between the carboxyl group (C-1) and the methyl group (H-10) via C-2. H-7 (δ_H_ 3.31) correlated with C-8 (δ_C_ 24.3), C-9 (δ_C_ 24.3), C-6 (δ_C_ 39.8), and C-5 (δ_C_ 148.1) (Figure 3), indicating that the *gem*-dimethyl fragment (CH_3_-8, CH_3–_9, C-6) is linked to C-5, and H-7 (δ_H_ 3.31) is attached to C-6 (δ_C_ 39.8). NOE correlations between H-3 (δ_H_ 6.38) and H-10 (δ_H_ 1.93) indicated that the double bond between C-2 and C-3 adopts a *Z* configuration. Based on these analyses, compound **1** was identified as (2*Z*,4*E*)-7-hydroxy-2,6,6-trimethylhepta-2,4-dienoic acid and named as diaporterpene D (**1**).

**TABLE 1:** ^1^H (600 MHz) and ^13^C (150 MHz) NMR data of **1**-**3** in methanol-*d*_4_.

No	1		2		3	
	δ_*C*_, type	δ_*H*_, (*J* in Hz)	δ_*C*,_ type	δ_*H*,_ (*J* in Hz)	δ_*C*,_ type	δ_*H*,_ (*J* in Hz)
1	172.5, C		181.3, C		181.1, C	
2	127.8, C		46.6, C		46.0, C	
3	140.4, CH	6.38, d (10.9)	150.5, CH	6.29, d (15.4)	144.3, CH	6.03, d (15.5)
4	126.8, CH	7.06, dd (15.6, 11.0)	123.8, CH	6.44, dd (15.4, 11.1)	126.8, CH	7.06, dd (15.5, 11.1)
5	148.1, CH	5.88, d (15.6)	140.9, CH	7.15, d (11.1)	137.6, CH	6.29, d (11.1)
6	39.8, C		126.8, C		129.1, C	
7	72.1, CH_2_	3.31, s	172.9, C		173.9, C	
8	24.3, CH_3_	1.03, s	25.8, CH_3_	1.33, s	25.7, CH_3_	1.30, s
9	24.3, CH_3_	1.03, s	25.8, CH_3_	1.33, s	25.7, CH_3_	1.30, s
10	21.1, CH_3_	1.93, s	12.9, CH_3_	1.91, s	21.3, CH_3_	1.93, s

Diaporterpene E (**2**) is a colorless oil. It was separated using chiral chromatography (Ultimate Amy-SR column, water-Acetone = 85/15, flow rate = 3 mL/min, Supplementary Figure S4). HR-ESIMS revealed a quasi-molecular ion peak at *m/z* 221.0783 [M+Na]^+^ (calcd for C_10_H_14_O_4_Na, 221.0784), confirming its molecular formula as C_10_H_14_O_4_, with an unsaturation degree of 4. The structure was elucidated using ^1^H NMR, ^13^C NMR, and HSQC spectra, revealing two carbonyl groups (δ_C_ 181.3, 172.9), two double bonds [δ_H_ 6.44 (H-4, dd, *J* = 15.5, 11.1 Hz, 1H), δ_C_ 123.8; δ_H_ 6.29 (H-3, d, *J* = 15.4 Hz, 1H), δ_C_ 150.5; δ_H_ 7.15 (H-5, d, *J* = 11.1 Hz, 1H), δ_C_ 140.9, δ_C_ 126.8], two quaternary carbons (δ_C_ 46.6, 126.8), and three methyl groups [δ_H_ 1.91 (H-10, s, 3H), δ_C_ 12.9; δ_H_ 1.33 (H-8,9, s, 6H), δ_C_ 25.8] (Table 1). The ^1^H-^1^H COSY spectrum and ^1^H NMR data indicated the presence of a 1,3-diene fragment. A comparison of the NMR data for compounds **1** and **2** reveals that **2** contains an additional carboxyl group and lacks the methylene alcohol fragment. This indicates that in compound **2**, the methylene alcohol group has been oxidized to a carboxylic acid. In the HMBC spectrum, the correlation of CH_3_-8/9 (δ_H_ 1.33) with C-1 (δ_C_ 181.3), C-2 (δ_C_ 46.6), and C-3 (δ_C_ 150.5) further confirms the oxidation of the methylene alcohol group to a carboxylic acid. Additionally, CH_3_-10 (δ_H_ 1.91) showed correlations with C-7 (δ_C_ 172.9), C-6 (*δ _C_* 126.8), and C-5 (*δ_C_* 140.9), establishing that the methyl group at H-10 is connected to the carboxyl group at C-7 (δ_C_ 172.9) and the olefinic carbon at C-6 (δ_C_ 126.8). In the NOE spectrum, the correlation between H-4 (δ_H_ 6.44) and H-10 (δ_H_ 1.91) suggests that the double bond in compound **2** adopts an *E* configuration. Thus, compound **2** is identified as (2*Z*,4*E*)-2,6,6-trimethylhepta-2,4-dienedioic acid named as diaporterpene E (**2**).

Diaporterpene F (**3**) is a colorless oil and an isomer of **2**, which was successfully isolated via chiral chromatography on an Ultimate Amy-SR column (water-acetonitrile = 85/15, flow rate = 3 mL/min, Supplementary Figure S4). The molecular formula was determined as C_10_H_14_O_4_ based on the positive HR-ESIMS ions at *m/z* 221.0782 [M+Na]^+^ (calcd for C_10_H_14_O_4_Na, 221.0784), suggesting 4 degrees of unsaturation. The structure contains two carbonyl groups (δ_C_ 181.2, 173.9), two double bonds [δ_H_ 7.06 (H-4, dd, *J* = 15.5, 11.1 Hz, 1H) δ_C_ 126.8, δ_H_ 6.03 (H-3, d, *J* = 15.5 Hz, 1H) δ_C_ 144.3; δ_H_ 6.29 (H-5, d, *J* = 11.1 Hz, 1H) δ_C_ 137.6, δ_C_ 129.1], two quaternary carbons (δ_C_ 46.0, 129.1), and three methyl groups [δ_H_ 1.93 (H-10, s, 3H) δ_C_ 21.3, δ_H_ 1.30 (H-8, 9, s, 6H) δ_C_ 25.7] (Table 1). In the HMBC spectrum, CH_3_-10 (δ_H_ 1.93) showed correlations with C-7 (δ_C_ 173.9), C-6 (δ_C_ 129.1), and C-5 (δ_C_ 137.6), confirming that the methyl group at H-10 is connected to the carboxyl group at C-7 (δ_C_ 173.9) and the olefinic carbon at C-6 (δ_C_ 129.1) (Figure 3). The chemical shift of C-10 (δ_C_ 21.1) in **1** matches the chemical shift of C-10 (δ_C_ 21.3) in **3**, while the C-10 chemical shift in **2** is 12.9. This suggests that the terminal double bond in **3** has the same *Z* configuration as in **1**. Therefore, **3** is identified as (2*E*,4*E*)-2,6,6-trimethylhepta-2,4-dienedioic acid and named as diaporterpene F (**3**).

Diaporpolypropionate A (**7**) is a colorless oil. HR-ESIMS spectrum revealed a quasi-molecular ion peak at *m/z* 225.1499 [M-H]^–^ (calcd for C_13_H_21_O_3_, 225.1496), confirming its molecular formula as C_13_H_22_O_3_, with a degree of unsaturation of 3. The ^1^H NMR, ^13^C NMR, and HSQC spectra revealed one carbonyl carbon (δ_C_ 182.4), two conjugated double bonds [δ_H_ 5.82 (H-5, d, *J* = 10.7 Hz, 1H) / δ_C_ 128.3, δ_H_ 6.27 (H-6, dd, *J* = 15.1, 10.7 Hz, 1H) / δ_C_ 127.7, δ_H_ 5.51 (H-7, dd, *J* = 15.1, 8.3 Hz, 1H) / δ_C_ 136.2, δ_C_ 134.7], one quaternary carbon (δ_C_ 134.7), three methine groups [δ_H_ 3.55 (H-9, p, *J* = 6.5 Hz, 1H) / δ_C_ 72.4, δ_H_ 2.18 (H-8, m, 1H) / δ_C_ 45.9, δ_H_ 2.61 (H-2, p, *J* = 6.5, 5.9 Hz, 1H)/δ_C_ 39.4], one methylene group [δ_H_ 2.41 (H-3a, dd, *J* = 13.6, 7.3 Hz, 1H), 2.09 (H-3b, dd, *J* = 13.6, 7.5 Hz, 1H)/δ_C_ 45.2], and four methyl groups [δ_H_ 1.74 (H-11, s, 3H) / δ_C_ 16.3, δ_H_ 1.10 (H-13, H-10, overlap, m, 6H)/δ_C_ 20.8, 17.2, δ_H_ 1.03 (H-12, dd, *J* = 6.8, 2.5 Hz, 3H)/δ_C_ 16.8] (Table 2). Based on these data, further corroborated by the ^1^H-^1^H COSY spectrum, which reveals the connections H-5/H-6, H-6/H-7, H-12/H-8, H-8/H-9, and H-9/H-13, compound **7** contains both a 1,3-diene fragment and a 2-butanol fragment. In the HMBC spectrum, H-12 (δ_H_ 1.03) correlated with C-8 (δ_C_ 45.9), C-7 (δ_C_ 136.2), and C-9 (δ_C_ 72.4), confirming the connection of the 1,3-diene and 2-butanol fragments through the C-7-C-8 bond. Additionally, H-10 (δ_H_ 1.10) correlated with C-1 (δ_C_ 182.4), C-2 (δ_C_ 39.4), and C-3 (δ_C_ 45.2), while H-3 (δ_H_ 2.41, 2.09) correlated with C-1 (δ_C_ 182.4), C-2 (δ_C_ 39.4), C-11 (δ_C_ 16.3), and C-4 (δ_C_ 134.7), establishing the presence of a 2-methylpropanoic acid moiety and confirming the connection of the terminal methyl (C-3) to the diene fragment. NOE correlations between H-6 (δ_H_ 6.27) and H-12 (δ_H_ 1.03), as well as between H-6 and H-11 (δ_H_ 1.74), suggest that the double bond in **7** adopts an *E* configuration. However, the NOE data alone cannot determine the relative configurations of the three chiral centers in **7**, indicating that there are eight possible stereoisomers. Therefore, further studies are necessary to definitively determine the stereochemistry of **7**. Overall, the planar structure of **7** was confirmed as (4*E*,6*E*)-9-hydroxy-2,4,8-trimethyldeca-4,6-dienoic acid and named diaporpolypropionate A (**7**).

**TABLE 2:** ^1^H (600 MHz) and ^13^C (150 MHz) NMR data of **7** and **8** in methanol-*d*_4_.

No	7		8	
	δ_*C*,_ type	δ_*H*,_ (*J* in Hz)	δ_*C*,_ type	δ_*H*,_ (*J* in Hz)
1	182.4, C		181.3, C	
2	39.4, CH	2.61, p (6.5, 5.9)	41.6, CH	3.39, m
3	45.2, CH_2_	2.41, dd (13.6, 7.3), 2.09, dd (13.6, 7.5)	131.4, CH	5.39, d (9.3)
4	134.7, C		135.6, C	
5	128.3, CH	5.82, d (10.7)	137.1, CH	6.07, d (15.3)
6	127.7, C	6.27, dd (15.1, 10.7)	128.4, CH	5.63, dd (15.3, 7.5)
7	136.3, C	5.51, dd (15.1, 8.3)	37.3, CH_2_	2.28, m; 1.90, m
8	45.9, CH	2.18, m	41.6, CH	1.51, m
9	72.4, CH	3.55, p (6.5)	71.4, CH	3.66, m
10	17.2 or 20.8, CH_3_	1.10, overlap	19.1, CH_3_	1.22, d (8.7)
11	16.3, CH_3_	1.74, s	12.9, CH_3_	1.78, s
12	16.8, CH_3_	1.03, dd (6.8, 2.5)	14.7, CH_3_	0.88, d (7.0)
13	17.2 or 20.8, CH_3_	1.10, overlap	20.3, CH_3_	1.13, d (6.4)

Diaporpolypropionate B (**8**) is a colorless oil. The HR-ESIMS analysis revealed a quasi-molecular ion peak at *m/z* 225.1497 [M-H]^–^ (calcd for C_13_H_21_O_3_, 225.1496), confirming its molecular formula as C_13_H_22_O_3_ with an unsaturation degree of 3. Analysis of the ^1^H NMR, ^13^C NMR, and HSQC spectra revealed the structure to contain one carbonyl carbon (δ_*C*_ 181.3), two double bonds [δ_*H*_ 6.07 (H-5, d, *J* = 15.3 Hz, 1H) δ_*C*_ 137.1, δ_*H*_ 5.63 (H-6, dd, *J* = 15.3, 7.5 Hz, 1H) δ_*C*_ 128.4, δ_*H*_ 5.39 (H-3, dd, *J* = 9.3 Hz, 1H) δ_*C*_ 131.4, 135.6], one quaternary carbon (δ_*C*_ 135.6), three methine groups [δ_*H*_ 3.66 (H-9, m, 1H) δ_*C*_ 71.4, δ_*H*_ 1.51 (H-8, m, 1H) δ_*C*_ 41.6, δ_*H*_ 3.39 (H-2, m, 1H) δ_*C*_ 41.6], one methylene [δ_*H*_ 2.28 (H-7a, m, 1H), 1.90 (H-7b, m, 1H) δ_*C*_ 37.3], and four methyl groups [δ_*H*_ 1.78 (H-11, s, 3H) δ_*C*_ 12.9, δ_*H*_ 1.13 (H-13, d, *J* = 6.4 Hz, 3H) δ_*C*_ 20.3, δ_*H*_ 1.22 (H-10, d, *J* = 8.7 Hz, 3H) δ_*C*_ 19.1, δ_*H*_ 0.88 (H-12, d, *J* = 7.0 Hz, 3H) δ_*C*_ 14.7] (Table 2). Comparison of the NMR data reveals that compounds **8** and **7** are highly similar; however, the coupling constants and splitting patterns of the vinyl protons in **8** differ from those in **7**. Additionally, in the HMBC spectrum of **7**, the methylene (H-3) was correlated with the carbonyl carbon (C-1), whereas in **8**, the methylene (H-7) did not correlate with the carbonyl carbon (C-1), but the vinyl proton at H-3 did. These differences indicate that while both compounds share the same carbon chain backbone, the conjugated double bond in **8** begins at position 3. The NOE spectrum showed that H-6 (δ_*H*_ 5.63) was correlated with H-12 (δ_*H*_ 0.88) and H-1 (δ_*H*_ 1.78), confirming that the double bond in **8** adopts the *E* configuration. Like **7**, the stereochemical confirmation of **8** requires further investigation. Therefore, compound **8** is identified as (3*E*,5*E*)-9-hydroxy-2,4,8-trimethyldeca-3,5-dienoic acid and named diaporpolypropionate B (**8**).

The known compounds were identified as 4-(1-hydroxy-1-methylethyl)benzoic acid (**4**) (Yang et al., 2023), (*E*)-2,2,6-trimethyl-3,5-heptadienoic acid (**5**) (Bullivant and Pattenden, 1976), (4*S*)-*p*-Menth-1-ene-4,7-diol (**6**) (Miyazawa and Kumagae, 2001), by comparing their spectroscopic data with those reported in the literature. LC-HRMS analysis comparing the production levels of compounds **1**–**3**, **7**, and **8** in the wild-type and *phoE* mutant strains revealed that the levels of these compounds were significantly higher in the mutant strain than in the wild type (Supplementary Figure S5), and the NMR, IR, and HR-ESI-MS spectra of the new compounds can be found in Supplementary Figures S6–S42.

### 3.3 Biosynthetic pathway of isolated new compounds

The biosynthesis of diaporterpenes D-F (**1**–**3**) is proposed to initiate from geranyl pyrophosphate (GPP), formed *via* condensation of isopentenyl pyrophosphate (IPP) and dimethylallyl pyrophosphate (DMAPP). Subsequent dephosphorylation and enzymatic oxidations, likely mediated by cytochrome P450 monooxygenases, alcohol dehydrogenases, and carboxylases, introduce hydroxyl and carboxyl groups. Isomerization of double bonds and regioselective oxidations ultimately give rise to the structural diversity observed in compounds **1**–**3**. Further bioinformatic analysis was performed to investigate the BGCs responsible for the production of compounds **1**–**3**. Unfortunately, the specific BGCs could not be definitively identified at this stage. In future studies, we plan to employ heterologous expression and gene knockout strategies to confirm and characterize the corresponding BGCs.

**FIGURE 4:**
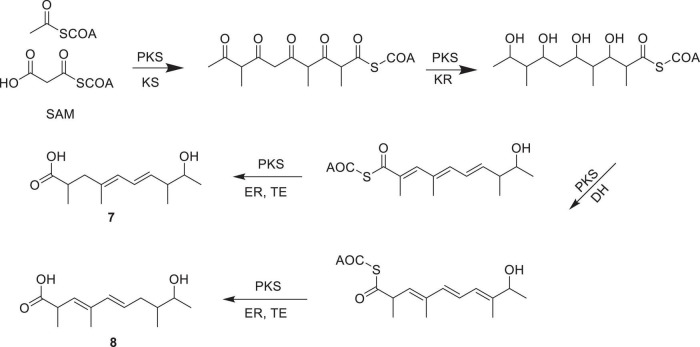
The biosynthetic pathway of polypropionate derivatives **7** and **8**.

The biosynthesis of compounds **7** and **8** proceeds *via* a polyketide pathway utilizing acetyl-CoA and malonyl-CoA as the starter and extender units, respectively. Successive condensations catalyzed by PKS modules, particularly ketosynthase (KS), drive chain elongation. Methyl branches are introduced by S-adenosylmethionine (SAM)-dependent methyltransferases. After chain assembly, selective modifications occur: ketoreductase (KR) reduces β-keto groups to β-hydroxy intermediates, while dehydratase (DH) and enoylreductase (ER) generate specific double bond patterns. Variation in double bond positions yields the two structural isomers, **7** and **8**. Finally, thioesterase (TE) catalyzes chain release, affording the final polypropionate products (Figure 4).

### 3.4 Cytotoxic and anti-inflammatory activities

**FIGURE 5:**
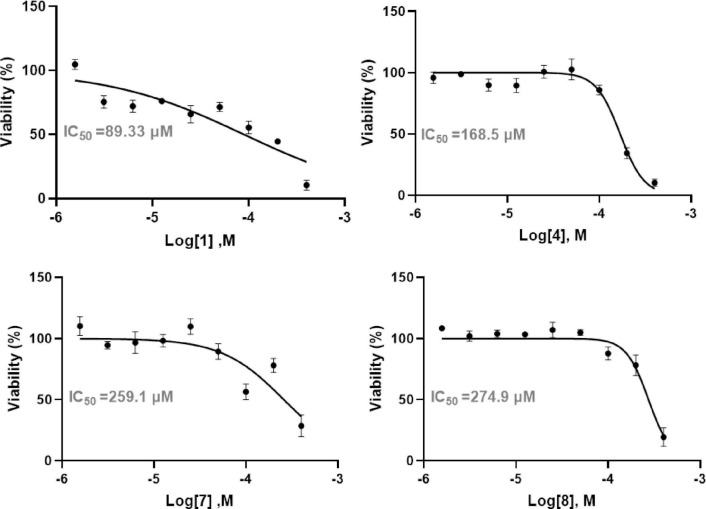
The anti-proliferative activity of compounds **1**, **2**, and **4**–**10** in human non-small cell lung cancer A549 cells.

We evaluated the cytotoxicity and anti-inflammatory effects of compounds **1**, **4**, **7**, and **8** in human non-small cell lung cancer A549 cells. The remaining compounds were not tested due to insufficient sample quantities. The anti-proliferative activity was determined by calculating the IC_50_ values after 48 h of treatment. As illustrated in Figure 5, diaporterpene D (**1**) demonstrated the most potent cytotoxicity, with an IC_50_ of 89.33 μM. In contrast, the other compounds exhibited weaker cytotoxic effects, with IC_50_ values above 150 μM. These findings suggest that diaporterpene D (**1**) effectively inhibit the proliferation of A549 cells in a dose-dependent manner, while the remaining compounds displayed limited cytotoxicity under the same conditions.

**FIGURE 6:**
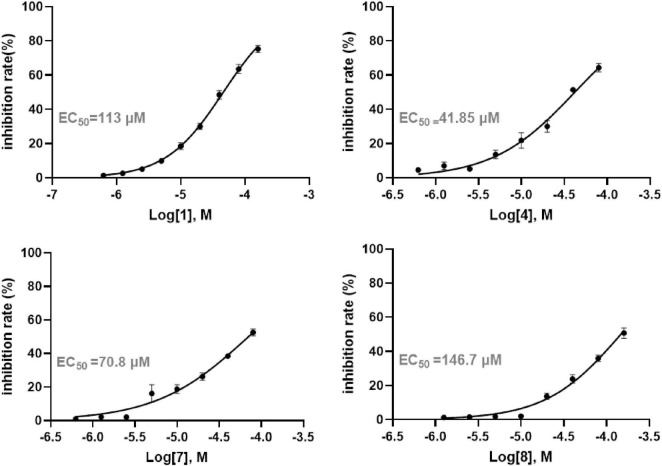
The anti-inflammatory activity of compounds **1**, **2**, and **4**–**10** in human non-small cell lung cancer A549 cells.

Following the determination of IC_50_ values, the anti-inflammatory potential of each compound was further assessed by quantifying IL-6 secretion in recombinant human interleukin-1β (rh IL-1β) induced A549 cells. The EC_50_ values from the dose-response curves demonstrated that **4** and diaporpolypropionate A (**7**) exerted strong inhibitory effects on IL-6 secretion, with EC_50_ values of 41.85 μM and 70.80 μM, respectively (Figure 6). Combined with cytotoxicity data, these findings indicate that both **4** and diaporpolypropionate A (**7**) are potent inhibitors of IL-6 secretion, showing significant anti-inflammatory activity while maintaining relatively low cytotoxicity. The dual properties of these compounds—potent anti-inflammatory effects combined with minimal cytotoxicity—highlight their potential as promising lead candidates for further research and drug development targeting inflammatory diseases.

## 4 Discussion

The method, known as the metabolic shunting strategy, involves inhibiting the biosynthesis of dominant metabolites to activate otherwise silent BGCs, and has been successfully applied in the discovery of novel secondary metabolites (Wei et al., 2021; Ning et al., 2024). For instance, the endophytic fungus *Penicillium dangeardii* predominantly produces rubratoxins at high yield; however, deletion of the key gene involved in rubratoxin biosynthesis resulted in the isolation of 23 new compounds from the mutant strain (Wei et al., 2021). Similarly, *Diaporthe* sp. SYSU-MS4722 primarily produces the xanthone phomoxanthone A at a high yield of approximately 3 g/L. Given this substantial production, it is likely that acetyl-CoA, the primary building block for polyketide biosynthesis, is largely consumed in the biosynthesis of phomoxanthone A, thereby limiting the availability of precursors for other secondary metabolic pathways. Inhibiting the production of such dominant metabolites in the wild-type strain can release key metabolic precursors (e.g., acetyl-CoA and malonyl-CoA), allowing them to be competitively utilized by alternative BGCs and consequently inducing the expression of cryptic gene clusters involved in the biosynthesis of novel natural products.

Given the limited knowledge regarding the biosynthetic mechanisms of monoterpenes in the *Diaporthe* genus, substantial opportunities remain for further investigation. Future investigations combining genome mining with the functional characterization of putative terpene synthase genes will be crucial for elucidating the enzymatic machinery underlying monoterpene biosynthesis (Scherlach and Hertweck, 2021). In addition, the application of transcriptomic and metabolomic analyses under various culture conditions may help uncover silent or conditionally expressed biosynthetic pathways. Functional validation through gene disruption, heterologous expression, and *in vitro* enzyme assays will further enable the reconstruction of complete biosynthetic routes (Hafner et al., 2021). These combined approaches are expected to expand our understanding of the structural diversity and biological functions of *Diaporthe*-derived monoterpenes and facilitate the discovery of novel bioactive compounds with potential pharmaceutical relevance.

## 5 Conclusion

*Diaporthe* sp. SYSU-MS4722 is known to produce monoterpenes but predominantly produces phomoxanthone A under standard laboratory conditions according to our previous study. To explore a broader range of monoterpenes, the mutant strain *Diaporthe* sp. SYSU-MS4722*△phoE*, which lacks the ability to produce phomoxanthone A, was cultivated in rice medium. This led to the identification of three new monoterpenes, diaporterpenes D-F (**1**–**3**), along with three known monoterpenes (**4**–**6**) and two novel polypropionate derivatives, diaporpolypropionate A (**7**) and diaporpolypropionate B (**8**). Compounds **1**, **4**, **7**, and **8** were evaluated for their cytotoxic and anti-inflammatory activities in human non-small cell lung cancer A549 cells. Compound **1** showed cytotoxicity with an IC_50_ value of 89.33 μM. Compounds **4** and **7** exhibited anti-inflammatory effects, as determined by an ELISA assay measuring IL-6 inhibition, with EC_50_ values of 41.85 and 70.80 μM, respectively.

## Data Availability

The data presented in this study are available via the ProteomeXchange Consortium at www.proteomexchange.org with the dataset identifier PXD065442.
