# Supplementary material for: Deletion of a polyketide synthase phoE induces monoterpenes production in ascidian-derived fungus Diaporthe sp. SYSU-MS4722
Source: Front Microbiol. 2025 Jul 3;16:1593027. doi: 10.3389/fmicb.2025.1593027 (PMC12269604; doi:10.3389/fmicb.2025.1593027)
Supplement: Supplementary file 1 [file Data_Sheet_1.docx]

**Genome Mining of Monoterpenes from Ascidian-Derived fungus *Diaporthe* sp. SYSU-MS4722**

Siwen Yuan ^1ǂ^, Shitao Zhuang ^2ǂ^, Yanhong Han ^2^, Yuhang Wen^1^, Senhua Chen ^3^, Lan Liu ^3^, Qifeng Lin ^3,4^, Yan Yan ^2,*^, Zhizeng Gao ^3,*^ and Qilin Wu ^1,*^

^1^ School of Bioengineering, Zunyi Medical University, Zhuhai 519041, China

^2^ Guangdong Provincial Engineering Research Center, The Fifth Affiliated Hospital, Sun Yat-Sen University, Zhuhai, 519000, Guangdong, China

^3^ School of Marine Sciences, Sun Yat-sen University, Zhuhai 519082, China

^4^ Shenzhen Lions King Hi-Tech Co., Ltd, Shenzhen 518000, China

^ǂ^ These authors contributed equally to this work

***Corresponding author.** **E-mail**: [wuqilin@zmuzh.edu.cn](mailto:wuqilin@zmuzh.edu.cn) (QLW); [gaozhizeng@mail.sysu.edu.cn](mailto:gaozhizeng@mail.sysu.edu.cn) (ZZG); [yanyan35@mail.sysu.edu.cn](mailto:yanyan35@mail.sysu.edu.cn) (YY)

Contents

[**Figure S1**. The predicted biosynthetic pathway of phomoxanthone A in our previous work 4](#_Toc200059499)

[**Figure S2**. Isolation of monoterpenes from *Diaporthe* sp. SYSU-MS4722 in our previous work 5](#_Toc200059500)

[**Figure S3**. Gene knockout of *phoE* in *Diaporthe* sp. SYSU-MS4722 in our previous wrok. 5](#_Toc200059501)

[**Figure S4**. Chiral separation of compounds **2** and **3**.. 5](#_Toc200059502)

[**Figure S5**. LC-HRMS analysis comparing the production levels of compounds **1**–**3, 7**, and **8** in the wild-type and *phoE* mutant strains 6](#_Toc200059503)

[**Figure S6**. ^1^H NMR spectrum for compound **1** (600 MHz, Methanol-*d*_4_) 6](#_Toc200059503)

[**Figure S7**. ^13^C NMR spectrum for compound **1** (150 MHz, Methanol-*d*_4_) 7](#_Toc200059504)

[**Figure S8**. HSQC spectrum for compound **1** 7](#_Toc200059505)

[**Figure S9**. HMBC spectrum for compound **1** 8](#_Toc200059506)

[**Figure S10**. ^1^H-^1^H COSY spectrum for compound **1** 8](#_Toc200059507)

[**Figure S11**. NOESY spectrum for compound **1** 9](#_Toc200059508)

[**Figure S12**. HR-ESI-MS spectrum for compound **1** 9](#_Toc200059509)

[**Figure S13**. ^1^H NMR spectrum for compound **2** (600 MHz, Methanol-*d*_4_) 10](#_Toc200059510)

[**Figure S14**. ^13^C NMR spectrum for compound **2** (150 MHz, Methanol-*d*_4_) 10](#_Toc200059511)

[**Figure S15**. HSQC spectrum for compound **2** 11](#_Toc200059512)

[**Figure S16**. HMBC spectrum for compound **2** 11](#_Toc200059513)

[**Figure S17**. NOESY spectrum for compound **2** 12](#_Toc200059514)

[**Figure S18**. HR-ESI-MS spectrum for compound **2** 12](#_Toc200059515)

[**Figure S19**. ^1^H NMR spectrum for compound **3** (600 MHz, Methanol-*d*_4_) 13](#_Toc200059516)

[**Figure S20**. ^13^C NMR spectrum for compound **3** (150 MHz, Methanol-*d*_4_) 13](#_Toc200059517)

[**Figure S21**. ^1^H^-1^H COSY spectrum for compound **3** 14](#_Toc200059518)

[**Figure S22**. HSQC spectrum for compound **3** 14](#_Toc200059519)

[**Figure S23**. HMBC spectrum for compound **3** 15](#_Toc200059520)

[**Figure S24**. HR-ESI-MS spectrum for compound **3** 15](#_Toc200059521)

[**Figure S25**. ^1^H NMR spectrum for compound **7** (600 MHz, Methanol-*d*_4_) 16](#_Toc200059522)

[**Figure S26**. ^13^C NMR spectrum for compound **7** (150 MHz, Methanol-*d*_4_) 16](#_Toc200059523)

[**Figure S27**. ^1^H-^1^H COSY spectrum for compound **7** 17](#_Toc200059524)

[**Figure S28**. HSQC spectrum for compound **7** 17](#_Toc200059525)

[**Figure S29**. HMBC spectrum for compound **7** 18](#_Toc200059526)

[**Figure S30**. NOESY spectrum for compound **7** 18](#_Toc200059527)

[**Figure S31**. ECD spectrum for compound **7** 19](#_Toc200059528)

[**Figure S32**. IR spectrum for compound **7** 19](#_Toc200059529)

[**Figure S33**. HR-ESI-MS spectrum for compound **7** 20](#_Toc200059530)

[**Figure S34**. ^1^H NMR spectrum for compound **8** (600 MHz, Methanol-*d*_4_) 20](#_Toc200059531)

[**Figure S35**. ^13^C NMR spectrum for compound **8** (150 MHz, Methanol-*d*_4_) 21](#_Toc200059532)

[**Figure S36**. HSQC spectrum for compound **8** 21](#_Toc200059533)

[**Figure S37**. HMBC spectrum for compound **8** 22](#_Toc200059534)

[**Figure S38**. ^1^H-^1^H COSY spectrum for compound **8** 22](#_Toc200059535)

[**Figure S39**. NOESY spectrum for compound **8** 23](#_Toc200059536)

[**Figure S40**. IR spectrum for compound **8** 23](#_Toc200059537)

[**Figure S41**. IR spectrum for compound **8** 24](#_Toc200059538)

[**Figure S42**. HR-ESI-MS spectrum for compound **8** 24](#_Toc200059539)

[**Reference** 25](#_Toc200059540)

**Figure S1**. The predicted biosynthetic pathway of phomoxanthone A in our previous work[1]

**Figure S2**. Isolation of monoterpenes from *Diaporthe* sp. SYSU-MS4722 in our previous work[2]


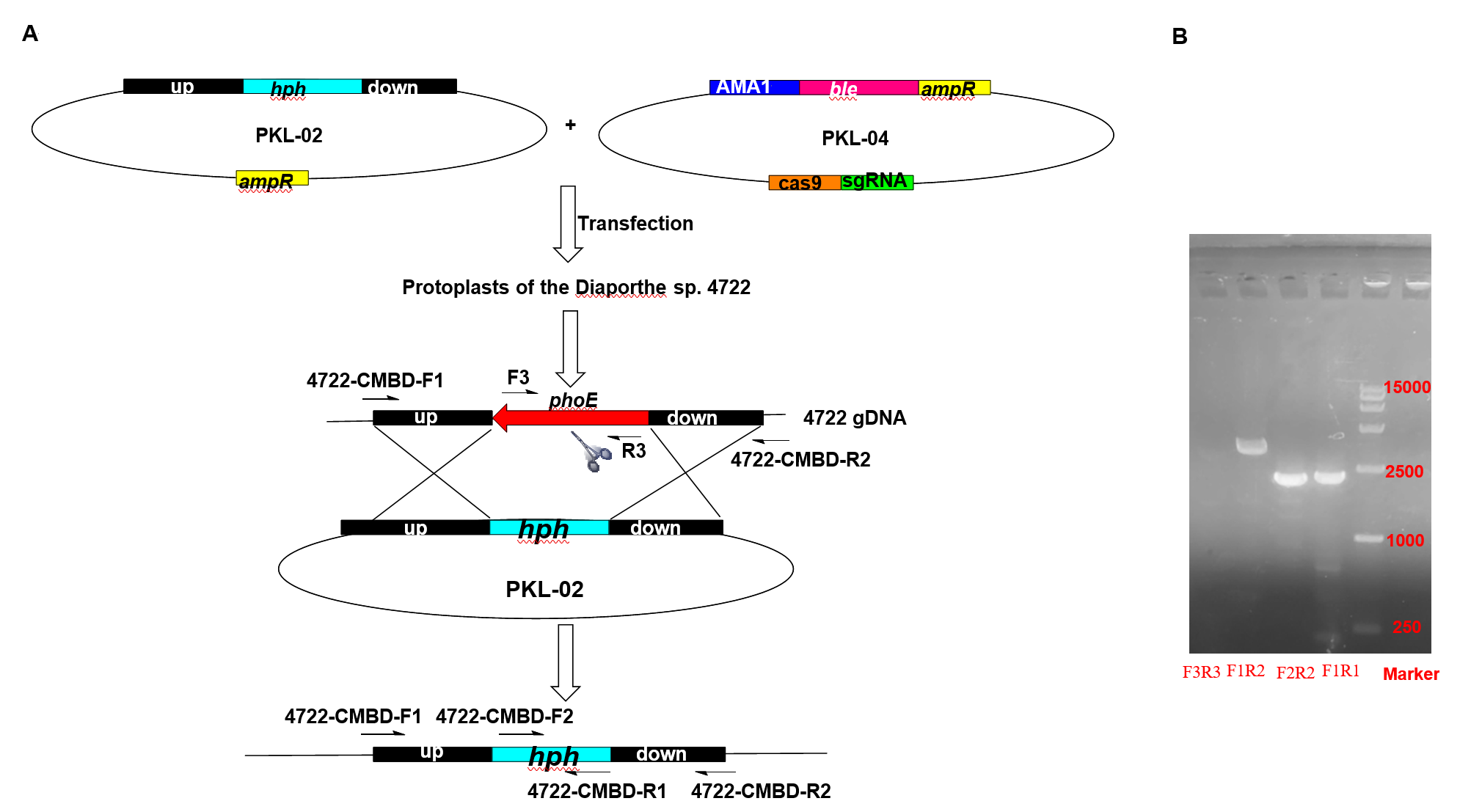


# **Figure S3**. Gene knockout of *phoE* in *Diaporthe* sp. SYSU-MS4722 in our previous wrok[1]. (A) The diagram of gene knockout of *phoE* and *hph* as the selective marker. (B) PCR analysis confirmed that the *phoE* was successfully disrupted.

**Figure S4**. Chiral separation of compounds **2** and **3** (Ultimate Amy-SR, MeCN/H_2_O = 15/85, flow rate = 3 mL/min)

**Figure** **S5.** LC-HRMS analysis comparing the production levels of compounds **1–3, 7**, and **8** in the wild-type and phoE mutant strains.

# **Figure S6**. ^1^H NMR spectrum for compound **1** (600 MHz, Methanol-*d*_4_)

# **Figure S7**. ^13^C NMR spectrum for compound **1** (150 MHz, Methanol-*d*_4_)

# **Figure S8**. HSQC spectrum for compound **1**

# **Figure S9**. HMBC spectrum for compound **1**

# **Figure S10**. ^1^H-^1^H COSY spectrum for compound **1**

# **Figure S11**. NOESY spectrum for compound **1**

# **Figure S12**. HR-ESI-MS spectrum for compound **1**

# **Figure S13**. ^1^H NMR spectrum for compound **2** (600 MHz, Methanol-*d*_4_)

# **Figure S14**. ^13^C NMR spectrum for compound **2** (150 MHz, Methanol-*d*_4_)

# **Figure S15**. HSQC spectrum for compound **2**

# **Figure S16**. HMBC spectrum for compound **2**

# **Figure S17**. NOESY spectrum for compound **2**

# **Figure S18**. HR-ESI-MS spectrum for compound **2**

# **Figure S19**. ^1^H NMR spectrum for compound **3** (600 MHz, Methanol-*d*_4_)

# **Figure S20**. ^13^C NMR spectrum for compound **3** (150 MHz, Methanol-*d*_4_)

# **Figure S21**. ^1^H^-1^H COSY spectrum for compound **3**

# **Figure S22**. HSQC spectrum for compound **3**

# **Figure S23**. HMBC spectrum for compound **3**

# **Figure S24**. HR-ESI-MS spectrum for compound **3**

# **Figure S25**. ^1^H NMR spectrum for compound **7** (600 MHz, Methanol-*d*_4_)

# **Figure S26**. ^13^C NMR spectrum for compound **7** (150 MHz, Methanol-*d*_4_)

# **Figure S27**. ^1^H-^1^H COSY spectrum for compound **7**

# **Figure S28**. HSQC spectrum for compound **7**

# **Figure S29**. HMBC spectrum for compound **7**

# **Figure S30**. NOESY spectrum for compound **7**


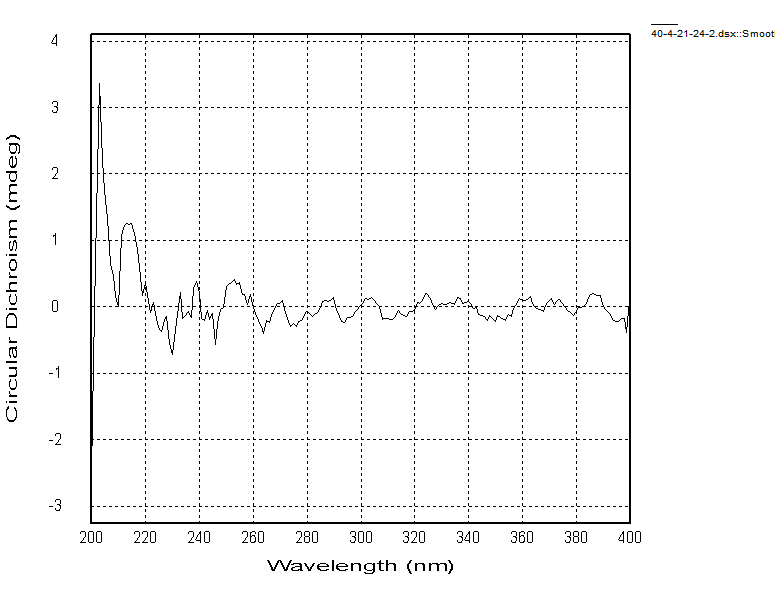


# **Figure S31**. ECD spectrum for compound **7**

# **Figure S32**. IR spectrum for compound **7**

# **Figure S33**. HR-ESI-MS spectrum for compound **7**

# **Figure S34**. ^1^H NMR spectrum for compound **8** (600 MHz, Methanol-*d*_4_)

# **Figure S35**. ^13^C NMR spectrum for compound **8** (150 MHz, Methanol-*d*_4_)

# **Figure S36**. HSQC spectrum for compound **8**

# **Figure S37**. HMBC spectrum for compound **8**

# **Figure S38**. ^1^H-^1^H COSY spectrum for compound **8**

# **Figure S39**. NOESY spectrum for compound **8**

# **Figure S40**. IR spectrum for compound **8**


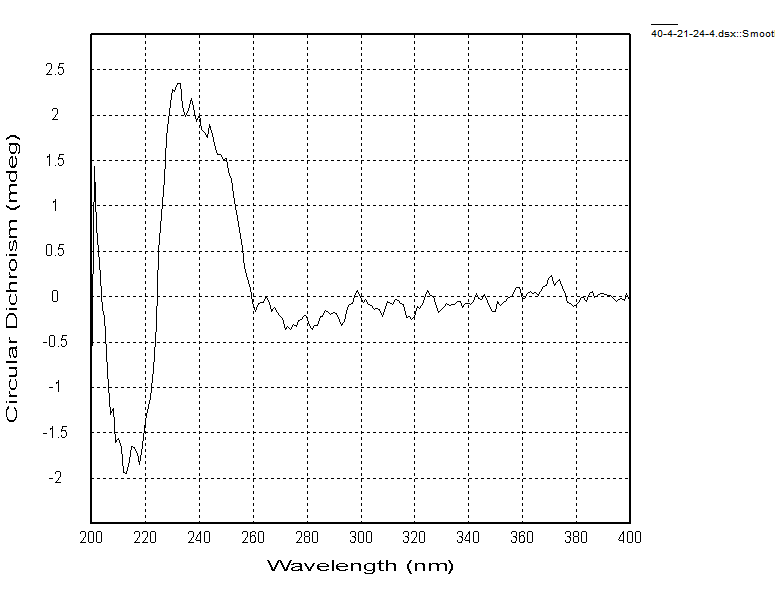


# **Figure S41**. IR spectrum for compound **8**

# **Figure S42**. HR-ESI-MS spectrum for compound **8**

# **Reference**

1. Yuan, S. W.; Chen, S. H.; Guo, H.; Chen, L. T.; Shen, H. J.; Liu, L.; Gao, Z. Z., Elucidation of the complete biosynthetic pathway of phomoxanthone A and identification of a para-para selective phenol coupling dimerase. *Org Lett.* **2022,** *24*, 3069-3074.

2. Zhai, G.; Chen, S.; Shen, H.; Guo, H.; Jiang, M.; Liu, L., Bioactive monoterpenes and polyketides from the ascidian-derived fungus *Diaporthe* sp. SYSU-MS4722. *Mar. Drugs* **2022,** *20*, 553.
